# Association Between Serum Calcium and the Prevalence of Hypertension Among US Adults

**DOI:** 10.3389/fcvm.2021.719165

**Published:** 2021-11-29

**Authors:** Yang Hua, Heng-li Liu, Jin-Yu Sun, Xiang-Qing Kong, Wei Sun, Ya-Qing Xiong

**Affiliations:** ^1^Department of Cardiology, The First Affiliated Hospital of Nanjing Medical University, Nanjing, China; ^2^Department of Cardiology, Geriatric Hospital of Nanjing Medical University, Nanjing, China

**Keywords:** association, serum calcium, hypertension, multivariable logistic regression, S-curve

## Abstract

**Background:** Hypertension is a significant risk factor of cardiovascular diseases, posing a serious threat to global health. Calcium plays an important role in regulating body homeostasis. The association of calcium with hypertension remains uncertain in the general population.

**Methods and Results:** Cross-sectional data from the 2007–2018 National Health and Nutrition Examination Survey (NHANES) were analyzed. Adjusted multivariable logistic regression analysis and restricted cubic spline were used to investigate the association of serum calcium with the prevalence of hypertension. A total of 26,778 participants were included. The increase in calcium levels showed a positive association with the prevalence of hypertension in all three models with ORs of 1.347 (1.249–1.454), 1.522 (1.401–1.654), and 1.438 (1.306–1.583). The further subgroup analysis demonstrated a robust trend across all categories by sex, age, race, BMI, and eGFR. The restricted cubic spline plot exhibited an S-curve relationship between calcium and hypertension.

**Conclusion:** Our cross-sectional study demonstrated a positive association between higher serum calcium level and the prevalence of hypertension. Our findings highlighted serum calcium level in hypertensive patients.

## Introduction

Hypertension is a common medical condition defined as systolic blood pressure (BP) ≥140 mmHg and/or diastolic BP ≥90 mmHg (1). The prevalence of hypertension has been consistently increasing in the past decades, especially in low- and middle-income countries, posing a serious threat to global health (2). Growing epidemiological evidence has confirmed the substantial influence of lifestyle interventions on blood pressure, including physical exercise, dietary patterns, and body weight management beyond genetic endowment (3).

As one of the most abundant mineral elements broadly involved in diverse body activities, the role of calcium in hypertension has received much attention as well as other cations. Nevertheless, unlike the normal recognized recommendation of sodium restriction and potassium intake for the dietary prevention of hypertension, previous studies that examined the association between serum calcium and hypertension have shown contradictory results (4–6). Some studies reported calcium to be associated with a higher risk of hypertension, whereas some shown an inverse or null effect. Most evidence from previous studies lack strong credibility due to the small sample size, incomplete adjustment of confounding factors, or excessive experimental extrapolation (5, 7).

Approximately 50% of serum calcium is in the ionized form, while 40% in the bound form mainly to albumin, and 10% is bound to anions (8). Serum total calcium is the total sum of three forms and is least affected by physiological changes or varieties in measurement. Therefore, total serum calcium is routinely used in clinical practice to represent calcium status in the human body (9). In this context, by using data from a large representative US population, we performed a cross-sectional study to investigate the association between calcium and the prevalence of hypertension.

## Materials and Methods

### Data Source and Study Population

Our study was a cross-sectional study. National Health and Nutrition Examination Survey (NHANES) is a public database recording the health and nutritional status among the US population and is published every 2 years (https://www.cdc.gov/nchs/nhanes/index.htm). A sample of subjects was selected and interviewed by using a stratified cluster sampling method to ensure the representativeness. We selected available data from the year cycle 2007–2008, 2009–2010, 2011–2012, 2013–2014, 2015–2016, 2017–2018. Individuals with full information on body measures, blood pressure, medical conditions, diabetes, smoking, alcohol consumption, dietary interview, and standard biochemistry profile were included. Based on previous literature (10, 11), the exclusion criteria were as followed: (1) pregnant individuals, (2) age <18 or ≥80, (3) estimated glomerular filtration rate (eGFR) < 60 ml/min/1.73m^2^, and (4) participants without calcium or phosphorus record. Notably, we excluded subjects with CKD [eGFR < 60 mL/min/1.73m^2^ (12)] to eliminate possible effects of CKD on calcium (13) and blood pressure (14). National Center for Health Statistics Research Ethics Review Board approved this study, and informed consent was obtained from all participants.

### The Definition of Hypertension

Blood pressure measurements were taken using a mercury sphygmomanometer according to standardized blood pressure measurement protocols recommended by American Heart Association at that time. After 5 min of seated rest, trained clinicians measured the blood pressure and repeated three times at the interval of 30 s. The mean of all available measurements was recorded. Hypertension was defined as (1) average systolic blood pressure ≥140 mmHg, (2) average diastolic blood pressure ≥90 mmHg, (3) current use of anti-hypertensive medications, (4) subjects with a self-reported hypertension (15–17). Moreover, we performed a sensitivity analysis by using a new cut-off value of 130/80 mmHg according to the American Heart Association (18).

### Covariates

Covariates related to hypertension were selected and controlled based on previously published studies (16, 19). We obtained age, sex (male and female), race/ethnicity (non-Hispanic white, non-Hispanic black, Mexican American, other Hispanic, and other races), and education levels (below high school, high school, and above high school) from the demographic questionnaire. Self-reported diabetes history (yes/no), smoking status (smoked at least 100 cigarettes in life or not), sodium intake, and alcohol consumption were acquired from dietary questionnaire, whereas total calcium (mg/dl), triglycerides (mg/dl), total cholesterol (mg/dl), albumin (g/L), serum phosphorus (mg/dl), and creatinine (mg/dl) were obtained from laboratory tests. Individuals smoking more than 100 cigarettes during their lifetime were considered smokers (20), and participants consuming at least 12 alcohol drinks per year were considered alcohol users (21). Body mass index (BMI) was calculated by dividing weight in kilograms by the square of their height in meters (kg/m^2^). Estimated glomerular filtration rate was estimated using the Modification of Diet in Renal Disease (MDRD) equation incorporating age, sex, race, and serum creatinine in the equation (22).

### Statistical Analysis

Continuous variables were represented as mean ± standard deviation (normal distribution), median with interquartile range (skewed distribution), or percentages (categorical variables). Comparisons between the hypertensive and non-hypertensive groups were performed using the chi-square test (categorical variables), one-way ANOVA test (normal distribution), or Kruskal-Wallis test (skewed distribution). Importantly, to maximize statistical power and minimize bias caused by missing data, we applied multivariate multiple imputation strategies based on five replication and Markov chain Monte Carlo method to fill missing covariates (23, 24).

Total calcium was categorized into quartiles, and the lowest quartile was set as the reference group. Multivariable logistic regression analysis was used to estimate the correlation between total calcium and hypertension. Three multivariable adjustment models were used: crude model (model 1) adjusted for age and gender; model 2 additionally adjusted for race/ethnicity, education levels, BMI, diabetes history, smoking status, alcohol consumption, sodium intake, triglycerides, and total cholesterol levels; model 3 adjusted for variables in model 2 plus albumin, serum phosphorus, and eGFR. The odds ratios (ORs) with 95% confidence intervals (CIs) were calculated accordingly. Subgroup analyses were performed to examine whether the associations of total calcium and hypertension were consistent across categories of sex (male and female), age (18–80), race (Non-Hispanic White, Non-Hispanic Black, Mexican American, other Hispanic, and Other-race including Multi-racial), BMI (underweight, normal weight, overweight, class I obese, class II obese, and class III obese), and eGFR (<133 ml/min/1.73 m^2^, and ≥133 ml/min/1.73 m^2^) using fully adjusted model 3. To further illustrate the correlation between total calcium and hypertension, we also used a restricted cubic spline with five knots located at the 5^th^, 27.5^th^, 50^th^, 72.5^th^, and 95^th^ percentiles to flexibly model the underlying relationship. The median total calcium was chosen as the reference.

Moreover, we performed Spearman correlation analysis to assess the correlation of total calcium and systolic/diastolic blood pressure. Receiver operating characteristic curve (ROC curve) was drawn to show identification ability of calcium in the prevalence of hypertension. We divided participants into a training set and a testing set randomly at a ratio of 7:3. The training set was used to create a predictive model, whereas the testing set was used to evaluate the model performance by area under the curve (AUC), sensitivity, specificity, positive predictive value, and negative predictive value. A sensitivity analysis using the cut-off value of 130/80 mmHg was also performed to validate the association between total calcium and hypertension.

All statistical analysis was performed by R software version 3.6.1 (version 3.6.0; The R Foundation for Statistical Computing). *P*-value < 0.05 was considered statistically significant for all analyses.

## Results

### Characteristics of Study Population

We initially included 34,573 subjects with data from NHANES 2007–2018. After excluding participants who were pregnant (*n* = 372), age <18 or >80 (*n* = 2168), eGFR < 60 ml/min/1.73 m^2^ or missing (*n* = 3,536), and those with other missing values for covariates, a total of 26,778 participants were ultimately included for further analysis. In the overall population, the prevalence of hypertension was 49.6%, the median age was 46 (33–60) years, and the median total calcium was 9.4 mg/dl. There were significant differences between the hypertensive and non-hypertensive groups in age, sex, race/ethnicity, education levels, triglycerides, total cholesterol, eGFR, total calcium, diabetes mellitus, BMI, drinking, and smoking (all *P* < 0.05). Hypertensive participants tended to have higher level of triglycerides, cholesterol, and BMI, lower eGFR, more probability of co-existed diabetes mellitus, smoking, and drinking behaviors than non-hypertensive participants. The general baseline characteristics of all subjects in this study are presented in Table 1.

**Table 1:** Baseline characteristics of study population.

	**Overall**	**Non-hypertensive**	**Hypertensive**	***P***
Number	26,778	13,495	13,283	
Age	46.0 [33.0, 60.0]	38.0 [28.0, 50.0]	55.0 [43.0, 64.0]	<0.001
Gender (Female/Male)	13,526/13,252 (50.5/49.5)	7,325/6,170 (54.3/45.7)	6,201/7,082 (46.7/53.3)	<0.001
Race (%)				<0.001
Non-Hispanic White	10,483 (39.1)	5,328 (39.5)	5,155 (38.8)	
Non-Hispanic Black	5,524 (20.6)	2,200 (16.3)	3,324 (25.0)	
Mexican American	4,367 (16.3)	2,421 (17.9)	1,946 (14.7)	
Other Hispanic	3,005 (11.2)	1,605 (11.9)	1,400 (10.5)	
Other Races	3,399 (12.7)	1,941 (14.4)	1,458 (11.0)	
Education (%)				<0.001
Below high school	6,330 (23.6)	2,905 (21.5)	3,425 (25.8)	
High school	6,026 (22.5)	2,874 (21.3)	3,152 (23.7)	
Above high school	14,422 (53.9)	7,716 (57.2)	6,706 (50.5)	
Triglycerides (mg/dl)	121.0 [80.0, 188.0]	106.0 [71.0, 164.0]	138.0 [92.0, 210.0]	<0.001
Cholesterol (mg/dl)	190.0 [164.0, 218.0]	187.0 [163.0, 213.0]	194.0 [167.0, 223.0]	<0.001
eGFR (ml/min/1.73 m^2^)	114.2 [91.4, 143.1]	118.7 [97.2, 145.0]	109.2 [86.1, 140.6]	<0.001
Total calcium (mg/dl)	9.4 [9.2, 9.6]	9.4 [9.1, 9.6]	9.4 [9.2, 9.6]	<0.001
Diabetes = No/Yes (%)	23,629/3,149 (88.2/11.8)	12,838/657 (95.1/4.9)	10,791/2,492 (81.2/18.8)	<0.001
BMI (kg/m^2^)	28.5 [24.7, 33.2]	26.7 [23.4, 30.9]	30.3 [26.5, 35.1]	<0.001
Drinking = No/Yes (%)	24,109/2,669 (90.0/10.0)	12,272/1,223 (90.9/9.1)	11,837/1,446 (89.1/10.9)	<0.001
Smoking = No/Yes (%)	15,028/11,750 (56.1/43.9)	8,136/5,359 (60.3/39.7)	6,892/6,391 (51.9/48.1)	<0.001

*BMI, body mass index; eGFR, estimated glomerular filtration rate. Data are presented as percentages for categorical variables or median with interquartile range for continuous variables with skewed distribution*.

### Association of Calcium With Hypertension

The results of multivariable logistic regression analysis are reported in Table 2. When treating total serum calcium as a continuous variable, the increase in serum total calcium showed a positive association with the prevalence of hypertension in all three regression models. When fully adjusted for age, gender, race/ethnicity, education levels, BMI, diabetes history, smoking status, alcohol consumption, sodium intake, triglycerides, total cholesterol levels, albumin, eGFR, and serum phosphorus, increased calcium was significantly associated with the prevalence of hypertension (OR, 1.438; 95%CI, 1.306–1.583). When using the lowest quartile of total calcium as a reference, individuals in the second to the fourth quartile had a higher risk of hypertension after fully adjusting for potential confounding factors. The ORs with 95% CIs for hypertension across increasing quartiles were 1.088 (1.008, 1.175), 1.228 (1.129, 1.336), and 1.432 (1.309, 1.567) in fully adjusted model.

**Table 2:** Association of calcium with hypertension.

	**Crude model (Model 1)**		**Model 2**		**Model 3**	
	**Odds ratio**	***P*-value**	**Odds ratio**	***P*-value**	**Odds ratio**	***P*-value**
Total calcium	1.347 (1.249–1.454)	<0.001	1.522(1.401–1.654)	<0.001	1.438 (1.306–1.583)	<0.001
Q1	Ref.		Ref.		Ref.	
Q2	1.054 (0.982–1.132)	0.145	1.112 (1.032–1.198)	0.005	1.088 (1.008–1.175)	0.03
Q3	1.164 (1.08–1.254)	<0.001	1.271 (1.175–1.376)	<0.001	1.228 (1.129–1.336)	<0.001
Q4	1.361 (1.263–1.466)	<0.001	1.506 (1.39–1.632)	<0.001	1.432 (1.309–1.567)	<0.001

*Crude model (Model 1): We adjusted for age, and gender. Model 2: We adjusted for age, gender, race/ethnicity, education levels, BMI, diabetes history, smoking status, alcohol consumption, sodium intake, triglycerides, and total cholesterol levels. Model 3: We adjusted for age, gender, race/ethnicity, education levels, BMI, diabetes history, smoking status, alcohol consumption, sodium intake, triglycerides, total cholesterol levels, albumin, eGFR, and serum phosphorus*.

**Figure 1:**
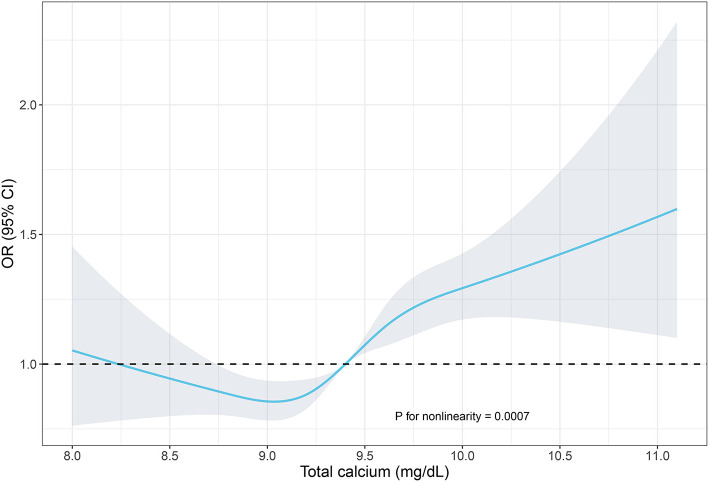
Restricted cubic spline of the association between serum total calcium and hypertension. The association was adjusted for age, gender, race/ethnicity, education levels, BMI, diabetes history, smoking status, alcohol consumption, sodium intake, triglycerides, total cholesterol levels, albumin, eGFR, and serum phosphorus. The median total calcium (9.4 mg/dl) was chosen as a reference. The plot showed a reduction of the risk within the lower range of serum calcium, which reached the lowest risk around 9.1 mg/dl and then increased thereafter. OR, odds ratio; CI, confidence intervals.

In Figure 1, we used restricted cubic spline to assess the relationship of total calcium and hypertension, and an S-shaped correlation was displayed. When setting the median calcium level as a reference, the prevalence of hypertension was increased in individuals with a higher serum total calcium.

**Figure 2:**
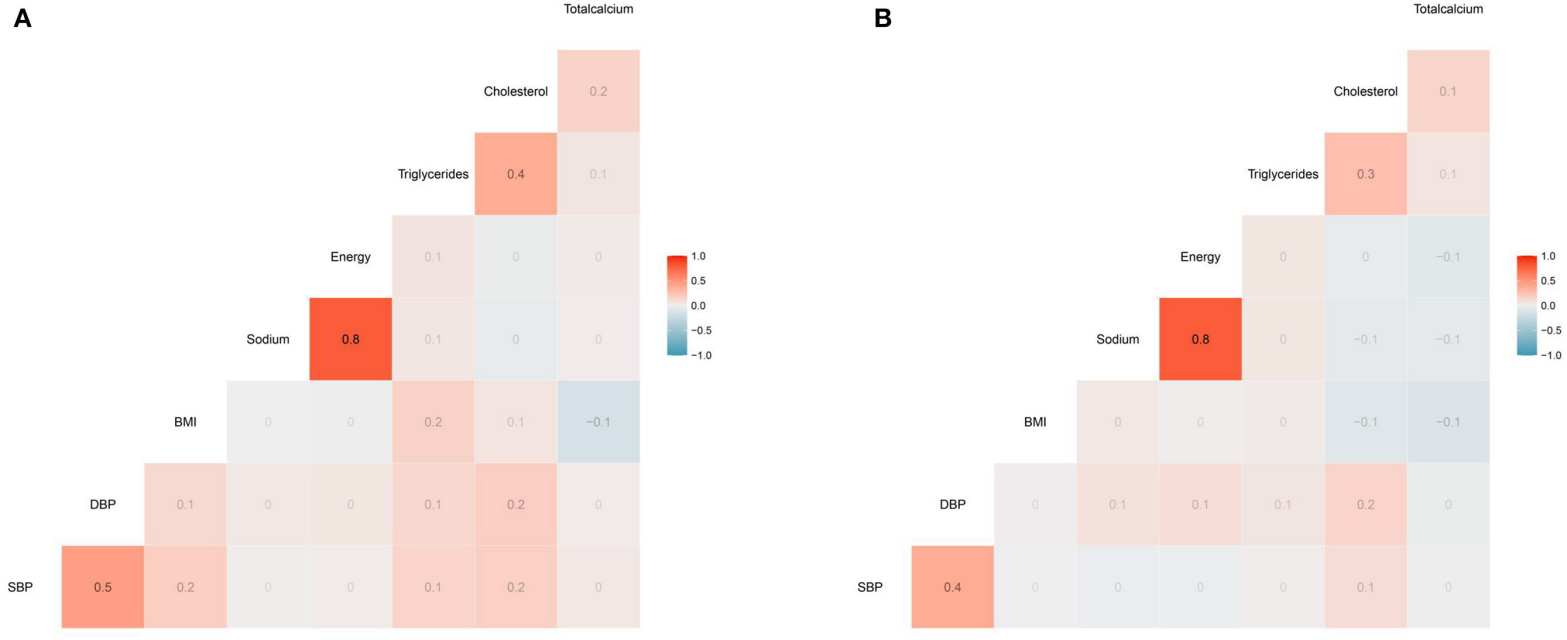
The heatmap of the correlation between covariates and SBP/DBP using the Spearman correlation analysis among participants **(A)** with anti-hypertensive medications **(B)** without anti-hypertensive medications. No direct correlation was found between total calcium and systolic pressure/diastolic pressure both in participants with and without anti-hypertensive medications. SBP, systolic blood pressure; DBP, diastolic blood pressure; BMI, body mass index.

Additionally, we performed Spearman correlation analysis to assess the association between varieties and systolic/diastolic blood pressure. To eliminate possible effects of anti-hypertensive medications, Spearman correlation analysis was performed, respectively. As shown in Figure 2, blood pressure was positively associated with BMI, triglycerides, and cholesterol. However, no direct correlation was found between total calcium and systolic pressure/diastolic pressure both in participants with and without anti-hypertensive medications.

Receiver operating characteristic curve was drawn to show identification ability of calcium in patients with hypertension. As shown in Supplementary Figure 1, serum calcium showed a poor identifying performance of the prevalence of hypertension, with an AUC of 0.53, sensitivity of 0.23, specificity of 0.81, positive predictive value of 0.55, and negative predictive value of 0.52 evaluated by the testing set.

### Subgroup Analysis

**Figure 3:**
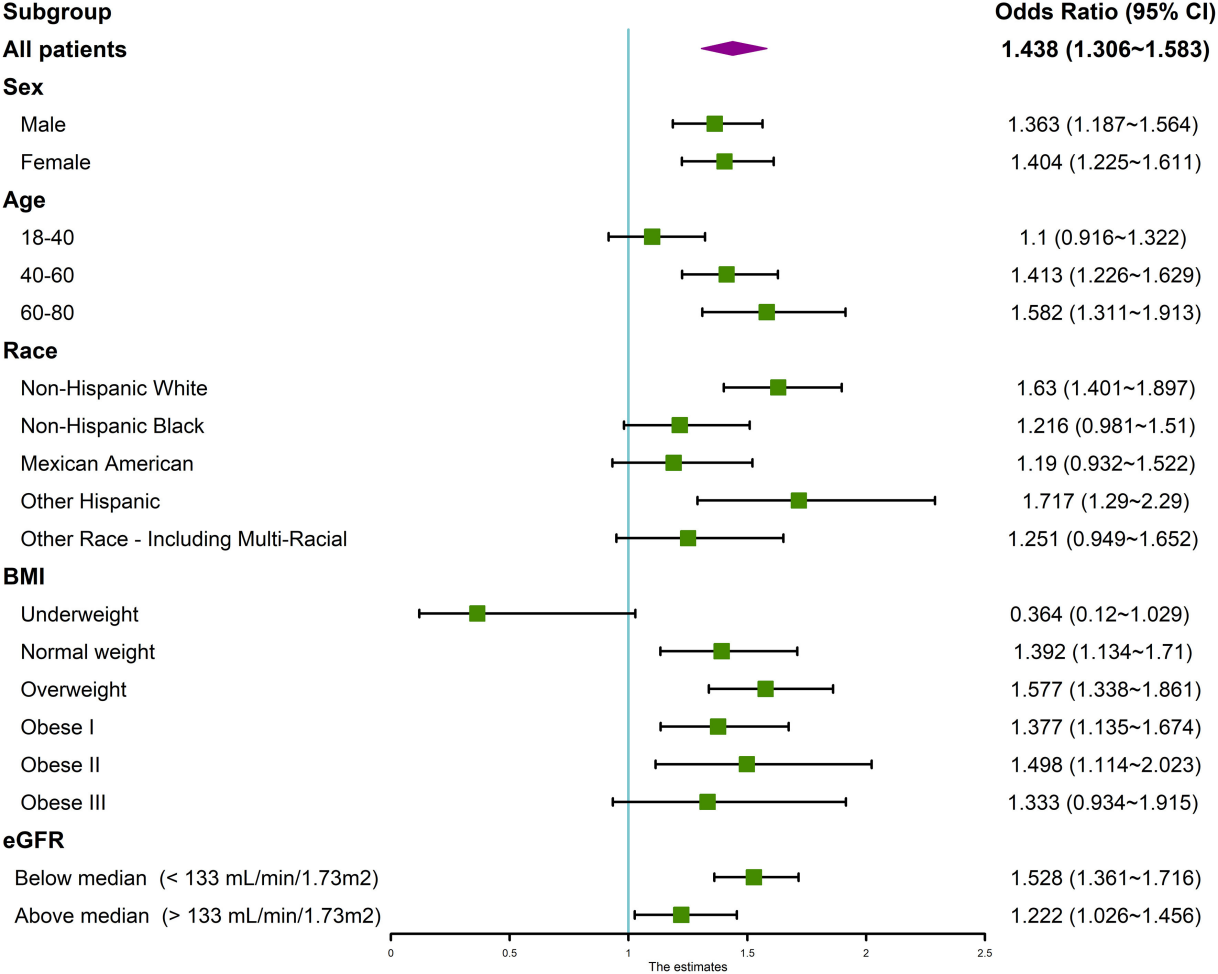
Forest plot of subgroup analysis of the association between serum calcium and hypertension. We used the ORs to evaluate the association between per 1 mg/dl increase in serum calcium with the prevalence of hypertension in the subgroup of sex, age, race, BMI, and eGFR. The association between serum calcium and hypertension remained significant across categories. The association was adjusted for age, gender, race/ethnicity, education levels, BMI, diabetes history, smoking status, alcohol consumption, sodium intake, triglycerides, total cholesterol levels, albumin, eGFR, and serum phosphorus. OR, odds ratio; CI, confidence intervals.

Subgroup analysis was subsequently performed based on categories of sex, age, race, BMI, eGFR. The association between serum calcium and hypertension remained significant across categories of sex, age, race, BMI, and eGFR. As shown in the forest plot (Figure 3), patients who were female, older, Non-Hispanic, other Hispanic, or multiracial, had a higher BMI were more disposed to hypertension.

To explore gender difference in the association of calcium with hypertension, we performed a subgroup analysis by sex and age (Table 3). Total serum calcium was found to be positively correlated to the prevalence of hypertension in both genders aged over 40. Conversely, there was no significant association of calcium with hypertension in young participants. When quartering total serum calcium level and using the lowest quartile of total calcium as a reference, a robust trend of greater prevalence of hypertension was observed with the increase in total serum calcium from the second to the fourth quartile among all age categories. Interestingly, the association of calcium with hypertension seemed to be stronger among females aged 40–60 compared with males at the same age. The ORs with 95% CIs for hypertension was 1.557 (1.282–1.894) for females and 1.24 (1.005–1.53) for males aged 40–60, respectively.

**Table 3:** Association of calcium with hypertension by sex and age using fully adjusted model.

**Model 3**					
**odds ratios (ORs) with 95% confidence intervals (CIs)**					
	**Total calcium**	**Q1**	**Q2**	**Q3**	**Q4**
Male	1.363 (1.187–1.564)				
18–40	1.157 (0.905–1.48)	Ref.	0.968 (0.795–1.177)	0.904 (0.739–1.106)	1.076 (0.872–1.328)
40–60	1.24 (1.005–1.53)	Ref.	1.04 (0.885–1.223)	1.133 (0.949–1.354)	1.303 (1.073–1.583)
60–80	1.598 (1.218–2.102)	Ref.	1.197 (0.969–1.481)	1.495 (1.179–1.903)	1.594 (1.23–2.074)
Female	1.404 (1.225–1.611)				
18–40	0.985 (0.745–1.304)	Ref.	0.981 (0.806–1.193)	1.028 (0.813–1.295)	1.144 (0.873–1.494)
40–60	1.557 (1.282–1.894)	Ref.	1.051 (0.897–1.232)	1.248 (1.041–1.497)	1.547 (1.277–1.875)
60–80	1.579 (1.213–2.062)	Ref.	1.207 (0.952–1.534)	1.541 (1.196–1.991)	1.528 (1.188–1.97)

*Model 3: We adjusted for age, gender, race/ethnicity, education levels, BMI, diabetes history, smoking status, alcohol consumption, sodium intake, triglycerides, total cholesterol levels, albumin, eGFR, and serum phosphorus*.

### Sensitivity Analysis

When the cut-off value was set as 130/80 mmHg, the prevalence of hypertension was 49.9%. Similarly, the ORs with 95% CIs for hypertension was 1.36 (1.26–1.468) in crude model (model 1), 1.544 (1.421–1.678) in model 2, and 1.457 (1.323–1.605) in model 3, respectively.

## Discussion

In our large, cross-sectional study among US adults, we observed a significant positive association of serum calcium level with the prevalence of hypertension. The association was independent of age, gender, race/ethnicity, education levels, BMI, diabetes history, smoking status, alcohol consumption, sodium intake, triglycerides, total cholesterol levels, albumin, eGFR, and serum phosphorus. When serum calcium level was analyzed as a categorical variable, the prevalence of hypertension for subjects in the highest calcium quartile was 1.45 times that of those in the lowest quartile. The association was robust in further subgroup analysis stratified by sex, age, race, BMI, and eGFR.

The relation between calcium intake and hypertension has long been a subject of debate since the 1980s. Most previous studies assessing the effects of calcium on blood pressure established a statistical correlation between low calcium level and a higher risk of elevated blood pressure (25–27). Moreover, numerous randomized controlled trials of calcium and vitamin D supplementation corroborated the antihypertensive role of calcium. Meta-analyses of randomized controlled trials demonstrated that calcium supplementation could decrease systolic blood pressure, with a mean difference of 2.5 mmHg (95% CI = 0.6–4.5) and 1.4 mmHg (95% CI= 0.72–2.15) in hypertensive and normotensive individuals, respectively (28, 29). Possible mechanisms may be that low plasmatic calcium concentration could stimulate the release of parathyroid hormone (PTH) (30) and parathyroid hypertensive factor (31), foster the synthesis of calcitriol (32), and activate RAAS system (33), which in return increase intracellular calcium concentration and lead to vasoconstriction, vascular resistance, and high blood pressure (34). Apart from this, both angiotensin II and PTH could provoke aldosterone secretion from adrenal gland (35), which could upregulate epithelial sodium channels (ENaC) in the principal cells of the collecting duct in the kidney, increase apical membrane permeability for Na+, facilitate Na+, and water reabsorption and thus, increase blood pressure (36). Collectively, the calcium deficiency hypothesis of hypertension populated and calcium supplementation was advocated in lowering blood pressure and delaying the onset of hypertension.

Nevertheless, some critics on the calcium deficiency hypothesis of hypertension arose with time, and the most quoted query came from Kaplan et al. (7). Kaplan stated that the theoretical construct was based on inconclusive extrapolation of experimental evidence obtained from epidemiologic, biochemical, and hemodynamic. Calcium deficiency should not be accepted as a mechanism responsible for hypertension, whereas excessive calcium or vitamin D supplementation should be used with caution. With the improvement of experimental design, expansion of sample size, and enhanced control of heterogeneity between trials, the role of calcium in the prevention and treatment of hypertension has again been controversial, and no specific consensus has been reached.

Our research was consistent with several previous studies examining the association of serum calcium and hypertension (5, 37–40). Charumathi and his associates documented that elevated serum total calcium levels were positively associated with hypertension after adjustments of serum albumin, 25(OH)D, serum phosphorus, and other confounders in a cross-sectional study of 12,405 US adults. Similarly, a study in the rural area of Northeast China aimed to assess the association of serum calcium and hypertension among adolescents aged 12–17 years showed that the multivariable ORs of hypertension among adolescents with serum calcium levels ≥2.53 mmol/L in comparison with serum calcium levels ≤ 2.37 mmol/L was 1.89 (1.41–2.53; *P* < 0.001) (5). Moreover, higher serum calcium levels were also positively associated with an average increase in SBP [4.22 (2.74–5.83; *P* < 0.001)] and DBP [2.23 (1.00–3.46; *P* < 0.001)], respectively. Kesteloot and his colleagues demonstrated that higher serum total calcium was positively associated with hypertension in both men and women after adjusting for serum creatinine and other confounders in a cross-sectional study of 4,167 men and 3,891 women in Belgium (39). Several longitudinal researches have also observed this association. XiaoYan Wu et al. conducted a prospective population-based study including 8,653 subjects with an average follow-up of 5.3 years in China (38). The results showed that the odd ratios for incident hypertension had a consistently increasing trend with increasing serum calcium concentration quartiles, indicating serum calcium level a significant risk of hypertension [1.37 (1.10, 1.70); 1.45 (1.17, 1.81); 2.18 (1.77, 2.68) for quartile 2, 3, and 4; quartile 1 set as a reference]. Cheng-Wai Chou and coworkers performed both cross-sectional analysis and longitudinal analysis among 27,364 community-dwelling participants in Taiwan during the period 2010–2016. They suggested that both serum calcium or albumin-corrected calcium was associated with an increased risk of hypertension (40).

Contrary to the positive correlation between serum calcium and hypertension at the clinical level, discrimination analysis using ROC curve failed to show a good predictive performance of hypertension by serum calcium with an AUC of 0.53 evaluated in the testing set. Interestingly, a satisfactory specificity of 0.81 was found, indicating that high serum calcium level might be a prominent phenotype of hypertension. Similarly, there was no apparent correlation witnessed by Spearman correlation analysis. As a matter of fact, hypertension is a long-term, systematic result of multiple factors, including genetic susceptibility, external environmental interference, sympathetic nervous system, hormones change, vascular abnormalities, etc. (41). So far, no single determining factor or biomarker of hypertension have been found (42). The multifactorial nature of pathogenesis of hypertension as well as lack of adjusted covariates in statistical analysis might partly explain for such confounding findings.

The mechanisms underlying the observed association between serum calcium and hypertension remain uncertain. Serum calcium may directly affect vasoconstriction by the influx of calcium into the smooth muscle of the artery, which enhances muscle contracture, increases vascular resistance, and therefore, leads to the development of hypertension (43). Additionally, calcium could indirectly promote hypertension by alteration in the extracellular binding of calcium (44), inducing insulin resistance (38), and interacting with other cations, especially sodium and potassium (45). Another possible mechanism lies in that subtle alterations in intracellular calcium may affect the secretion and action of hormones, such as the pressor action of catecholamines, angiotensin II, or aldosterone, which may target the blood pressure control centers and increase blood pressure levels (6). Moreover, calcium could mediate PTH release and several previous studies have found a positive correlation between PTH concentration and blood pressure (46, 47).

In our study, we observed a slightly stronger association between calcium and the prevalence of hypertension in females than males. A subsequent logistic regression analysis by sex and age found that the OR for hypertension was much greater in females aged 40–60 compared to males at the same age, which might explain for overall gender variance. To date, there was no report of such interesting findings in previous large, cross-sectional study. When we try to understand gender variance in the association between calcium and the prevalence of hypertension in middle-aged participants, we may inevitably encounter the influence of menopause in women. Menopause is a unique and natural process in women's lives during which sex hormones fluctuate and vast changes in metabolic state occur. It has been well-confirmed by epidemiological data that women had an obvious increase in blood pressure during menopause vs, age-matched men, which contributed to a greater prevalence of hypertension among women aged over 65 years in the US (48). The reduction of estrogen, a protective anti-hypertensive factor, may reduce the release of nitric oxide (NO), promote vascular remodeling process, increase endothelial dysfunction, and lead to the progression of hypertension (49, 50). It is reasonable to speculate that the metabolic state disparity between the two genders during such unique process of women would lead to variance in the association between calcium and hypertension. Although the beneficial role of adequate calcium has been addressed regarding bone metabolism, obesity, and even hypertension in middle-aged women (51, 52), a rising body of evidence linking calcium supplementation with adverse cardiovascular events such as coronary artery calcification, and cardiovascular mortality has risen to be a cause for concern (4). Our large, cross-sectional study suggested that total serum calcium might be a more significant risk factor for hypertension in middle-aged females. Given that calcium is only available to the body through dietary sources, cautions should be raised to blinded calcium supplementation especially in such populations. More specific basic and clinical researches are needed in the future.

There are several limitations to our study. First, though a large study sample was assessed, extensive international research composed of diverse ethnicities and regions was required to establish the association between calcium and hypertension better. Second, even if we have searched the literature and tried our best to adjust for potential confounders, unknown and complex confounders may exist, such as plasma renin activity (53), aldosterone (54), and anti-hypertensive medications interfering with our results. Third, concentrations of total calcium might be influenced by nutrition intake or alterations in biological factors (55, 56). For instance, menopausal status, use of calcium supplements, and use of osteoporosis medications may affect serum calcium level and cause bias. In addition, calcium was measured at baseline or at the initiation of the observation period without taking the changes in concentration into consideration in our study. Last but not least, the cross-sectional nature of the study limits making causal inferences.

## Conclusion

Serum total calcium levels were found to be positively associated with the prevalence of hypertension in a representative sample of US adults.

## Data Availability Statement

The datasets presented in this study can be found in online repositories. The names of the repository/repositories and accession number(s) can be found in the article/Supplementary Material.

## Author Contributions

WS, Y-QX, and X-QK conceived and designed the study. J-YS and H-lL analyzed the data. YH wrote the paper. All authors provided critical revisions of the manuscript and approved the final manuscript.

## Funding

This research was supported by Jiangsu Medical Science and Technique Development Foundation (H201638), the National Key Research and Development Program of China (No. 2019YFA0210100), and China International Medical Foundation (Z-2019-42-1908).

## Conflict of Interest

The authors declare that the research was conducted in the absence of any commercial or financial relationships that could be construed as a potential conflict of interest.

## Publisher's Note

All claims expressed in this article are solely those of the authors and do not necessarily represent those of their affiliated organizations, or those of the publisher, the editors and the reviewers. Any product that may be evaluated in this article, or claim that may be made by its manufacturer, is not guaranteed or endorsed by the publisher.

## Supplementary Material

Supplementary Figure 1 The ROC curve of the model performance in the testing set. All individuals were randomly divided into a training set and testing set at a ratio of 7:3. We used the training set to create a predictive model, whereas the testing set was used to evaluate the model performance. Serum calcium showed a poor identifying performance of the prevalence of hypertension, with an AUC of 0.53, sensitivity of 0.23, specificity of 0.81, positive predictive value of 0.55, and negative predictive value of 0.52. AUC: area under the curve.
