# Supplementary figures and images for: Association Between Serum Calcium and the Prevalence of Hypertension Among US Adults
Source: Front Cardiovasc Med. 2021 Nov 29;8:719165. doi: 10.3389/fcvm.2021.719165 (PMC8666532; doi:10.3389/fcvm.2021.719165)

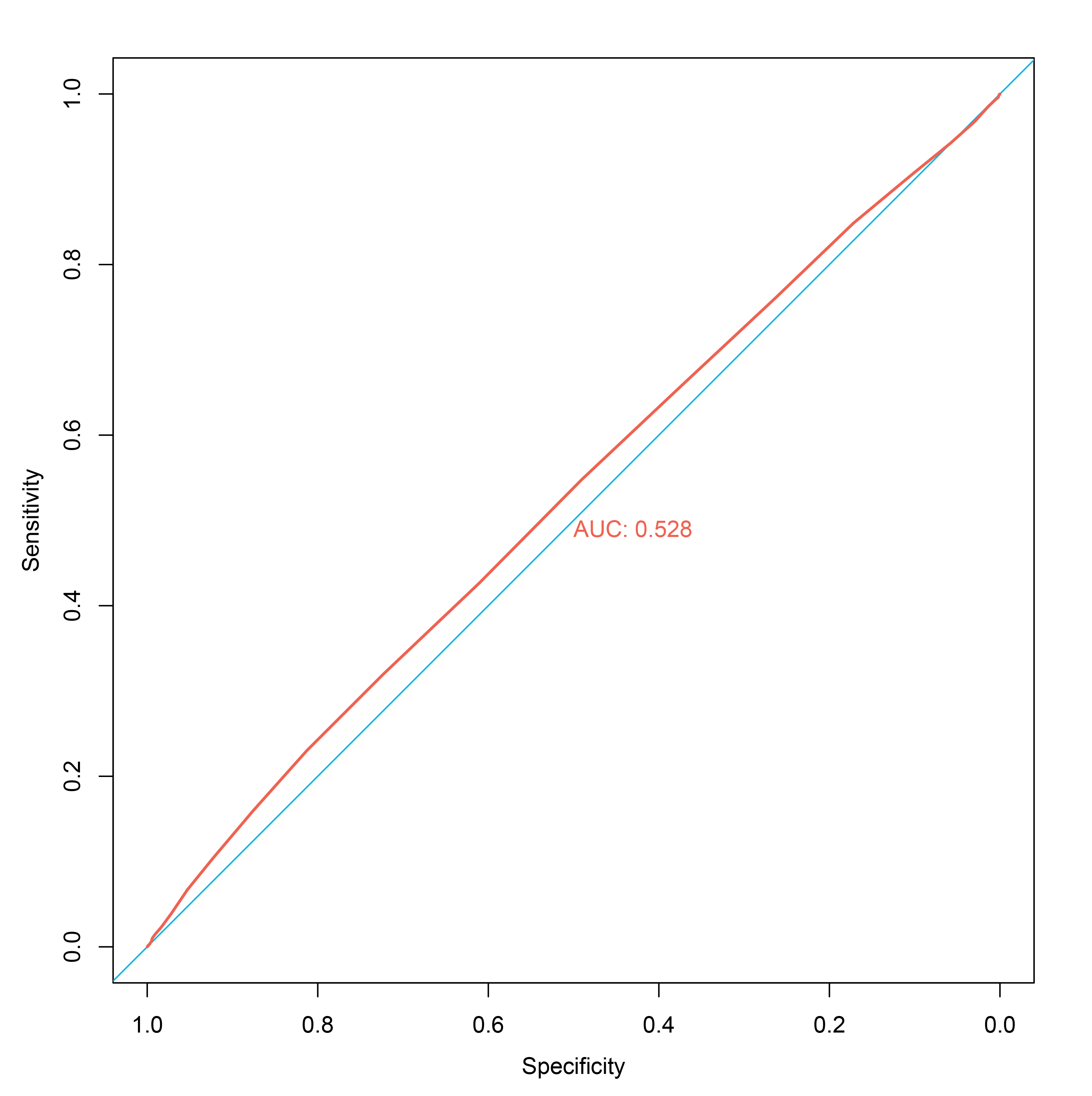

Supplement: Supplementary Figure 1 — The ROC curve of the model performance in the testing set. All individuals were randomly divided into a training set and testing set at a ratio of 7:3. We used the training set to create a predictive model, whereas the testing set was used to evaluate the model performance. Serum calcium showed a poor identifying performance of the prevalence of hypertension, with an AUC of 0.53, sensitivity of 0.23, specificity of 0.81, positive predictive value of 0.55, and negative predictive value of 0.52. AUC: area under the curve. [file Image_1.tif]
